# Upper Egypt experience in bladder preservation using concurrent chemoradiotherapy

**DOI:** 10.1186/1755-7682-6-21

**Published:** 2013-05-06

**Authors:** Ahmed M Maklad, Elsayed M Ali, Ashraf Elyamany, Emad Eldin Nabil

**Affiliations:** 1 Clinical Oncology Department, Sohag University, Sohag, Egypt; 2 Medical Oncology Department SECI, Assiut University, Assiut, Egypt

## Abstract

**Objective:**

To share our experience in bladder preservation in Upper Egypt, Assiut and Sohag Universities, using different treatment protocols. In Sohag study patients with operable muscle invasive bladder cancer were included and underwent transurethral resection followed by radiochemotherapy (5- fluorouracil and Cisplatin) for bladder preservation. In Assiut study after maximum safe resection of bladder tumor, patients received combined chemo-radiotherapy, 60 Gy of fractionated radiotherapy over 6 weeks, with Cisplatin and Gemcitabine.

**Results:**

In Sohag study the age of patients ranged from 35–72ys with Median 56 years, 24 patients were male (80%) and 6 patients were female (20%). In Assiut study the mean of age was 57.30 years, median 58.5 years with peak incidence in 7th decade (9 cases) then in 6th decade 7 cases (23.33%). Performance status was represented as following, 23 patients (76.6%) were scale 1 and seven patients (23.3%) were scale 2. In Assiut study, 90% of patients were disease free at the time of cystoscopic reevaluation. Of concern is that within 18 months of follow up in Assiut study, 7 of 27 (74%) complete responding patients have had local recurrence and 66.7% of all cases. The recurrence free survival in Sohag study at the median follow up (17 months) was 84% and at the end of follow up (30 months) was 70%. The overall survival at the median follow up was 95%, and at the end of follow up was 84%. The disease free survival in Assiut study was 66.7% and the overall Survival in Assiut study was 76.7.

**Conclusion:**

Three significant prognostic factors were detected for overall survival, performance status, tumor size and residual of tumor and two significant prognostic factors were detected for disease free survival, tumor size and residual of tumor in Assiut study. And it was nearly similar to that reported by Sohag study as they found the completeness of TUR and early stage of the tumor had the strongest impact in response to treatment.

## Introduction

Radical cystectomy has been considered the treatment of choice for non metastatic muscle invasive bladder cancer. This approach results in 90% local control at 5 years, and only 40% to 60% 5 year overall survival [1]. In addition, there is a high incidence of life altering morbidity after radical cystectomy, such as the need for urinary diversion. As a result, alternative, bladder sparing treatment approaches have been explored. Trimodality bladder preserving therapy, with combined transurethral resection, radiation, and chemotherapy, provides survival comparable with radical cystectomy. Five year survival with bladder preservation was 38% to 45% and 80% of the long term survivors maintained a functional bladder, with no apparent cost in terms of survival [2].

The majority of clinical trials in the past decade have used single agent 5–fluorouracil or Cisplatin for radiosensitization. In an attempt to improve safety and to increase efficacy, newer studies of multimodality therapy were done, including chemotherapeutic agents that have recently shown excellent activity in metastatic uroepithelial cancers, such as gemcitabine. Many phase II trials demonstrated that gemcitabine combined with cisplatin was well tolerated active regimen [3]. Our aim is to evaluate the efficacy and safety of combined chemo-radiotherapy after maximum resection of bladder tumor, in treatment of invasive bladder cancer, and to estimate response and survival rates in patients subjected to bladder conservation protocols. We will provide our experience using different treatment protocols. We will discuss 2 different studies with different bladder preservation approaches; both studies were done in Upper Egypt, the first study (group A) at Sohag University and the second study (group B) at Assiut University.

## Patients and methods

The first study, group (A), was performed in the clinical oncology department, faculty of medicine, Sohag University between the periods from September 2002 to April 2005.

30 patients with operable muscle invasive transitional cell carcinoma of bladder were included. They underwent transurethral resection followed by radiochemotherapy for bladder preservation.

Eligibility Criteria: Age, 18–75 years, ECOG (Eastern Cooperative Oncology Group) performance status 0–2 Transitional cell carcinoma of the urinary bladder, TNM stages (T2- T4a, No, Mo), Resectable disease, Prostatic urethral involvement with transitional cell carcinoma was allowed, if it was completely resected and no stromal invasion. No distant metastases,hemoglobin was equal or more than 10 g/dl, WBC equal or more than 3000 mm3 (neutrophil count equal or more than 1000/mm3 & platelets count equal or more than 100,000/mm3. bilirubin less more than 1.5 mg/dl, normal liver and renal function, creatinine was not more than 1.5 mg/dl. No prior systemic chemotherapy or pelvic radiotherapy was given.

### Treatment plan for group (A)

Concomitant radio-chemotherapy was delivered within 3 to 4 weeks after transurethral resection.

#### Phase I of radiochemotherapy treatment (weeks 1–3)

Radiotherapy was given by Linear accelerator (6 MV), twice daily fractions of external beam irradiation was given as follow, 1.6 GY was given to the pelvic field in the first fraction followed by an interfraction period of at least 4–6 hours, then, 1.5 GY was given to the bladder field in the second fraction, this treatment was given in days 1–5 & 8–12 & 15, 16 17. Chemotherapy was given combined with radiotherapy as following: 5-fluorouracil (400 mg/m2) was administered as a 24- hour’s infusion on days (1, 2, 3 and 15, 16, 17). Cisplatin (15 mg/m2) was administered as a 60- minutes infusion on days (1, 2, 3 -- 8, 9, 10 -- 15, 16, 17).

#### Phase II radiochemotherapy (weeks 8, 9)

1.5 GY was delivered to the pelvic field twice daily for 8 days, (day 1, 2, 3, 4, 5, 8, 9, 10). Both fractions were given to the pelvic field with a minimum 4 hours interval. The radiation dose delivered during phase II was 24 GY. Chemotherapy was given during phase II as follow: 5- fluorouracil (400 mg/m2) was given in days 1, 2, 3 and days 8, 9, 10. Cisplatin (15 mg/m2) was given in days 1, 2 and days 8, 9.

Total dose of radiotherapy delivered to the bladder after end of treatment was 64.30 GY. The maximum radiation dose reached to the posterior wall of the rectum and to the femoral heads did not exceed 55 GY and 45 GY, respectively.

The second study (Group B), this study was performed at Assiut University Hospital, during period from December 2004, to April 2006, 30 patients were enrolled in this trial.

Eligibility criteria: Patients with histopathologically proved invasive bladder cancer, (T2 – T3b, No – Mo). After maximum transurethral resection of bladder tumor, followed by second look cystoscopy for resection of any residual tumor at tumor bed after two weeks, or partial cystectomy. Karnofsky scale ≥ 70%. Adequate hematological, hepatic and renal functions. Adequate bladder capacity ≥ 350 c.c. this was evaluated under anesthesia.

Patients who previously received BCG or intravesical chemotherapy were excluded from the study.

### Treatment plan for group (B)

After complete resection of bladder tumor, patients received combined chemo-radiotherapy, 60 Gy of fractionated radiotherapy (200 cGy per setting) over 6 weeks with Cisplatin 75 mg/m2 every 3 weeks and Gemcitabine 300 mg/m2 D 1, 8 and 15 every 3 weeks for 2 cycles.

During the first phase, a fractionated dose of 4600 cGy/23 fractions was administered to bladder and pelvic lymphatics (L5-S1 to obturator foramen & 1.5 cm outside pelvic inlet), and the patient was instructed to be full bladder. Then the second phase in which 1400 cGy/7 fractions was given to bladder with safety margin 2 cm all around using one anterior open and 2 lateral wedged fields on linear accelerator (6 m.v. and 15 m.v.) according to separation and distribution of isodose curves. The patient was instructed to be empty bladder in second phase.

Complete clinical and radiological reevaluation was performed 4–6 weeks after the end of treatment and every 3 months for 18 months.

### Statistical methods of analysis

Statistical analysis of data was done by the statistical package for the social science (SPSS).

Survival analysis: Overall survival (OS) was calculated from the date of diagnosis to the date of death. Disease free survival (DFS) was calculated from the date of diagnosis to the date of treatment failure (recurrence of disease or distant metastasis). Kaplan-Meier life table was used to study OS and DFS.

Tests of significance: *T* test, Anova and Chi-square were used to study significance differences between variables.

## Results

In group (A), 30 patients with operable transitional cell carcinoma of the bladder underwent maximum transurethral resection followed by radio-chemotherapy as an initial treatment.

The age of patients in group A ranged from 35–72 y with Median age 56 years, 24 patients were male (80%) and 6 patients were female (20%). Performance status was represented as following, 23 patients (76.6%) were scale (1) and seven patients (23.3%) were scale (2) Table 1.

**Table 1:** Baseline patient characteristics for group (A) and (B)

**Characteristics**	**Group A**	**Group B**
	**(n= 30)**	**(n=30)**
median age “years”	56	57.3
Sex		
male	24 (80%)	21 (70%)
female	6 (20%)	9 (30%)
Performance status		
1	23 (76.6%)	29 (97%)
2	7 (23.3%)	1 (3%)
Transurethral resection		
complete	8 (26.6%)	25 (83.3%)
incomplete	22 (73.3%)	5 (16.7%)
Disease stage at presentation		
T2a	2 (7%)	2 (7%)
T2b	9 (30%)	25 (83%)
T3a	11 (37%)	2 (7%)
T3b	7 (23%)	1 (3%)
T4a	1 (3%)	-----
Grade		
I	0	9
II	18 (60%)	10 (33.3%)
III	12 (40%)	11 (36.7 %)
Bilharzial type		
Yes	0	13 (43.3%)
No	30	17 (56.6%)
Type of surgery		
Partial cystectomy	0	7 (23.3%)
TURT	30	23 (76.6%)

All patients underwent transurethral resection, 8 patients had complete TUR while in 22 patients incomplete TUR was done (26.6%, 73.3% respectively).

The T stages of the disease were distributed as follow, 2 patients were diagnosed as stage T2a (6.6%), 9 patients stage T2b (30%), 11 patients stage T3a (36.6%), 7 patient stage T3b (23.3%) and one patient stage T4a (3.3%).

18 patients (60%) were grade II and 12 patients (40%) were GIII. 13 patients (43.3%) their pathology was associated with bilharziasis.

Group (B): Thirty patients were included (21 men and 9 women) with a mean age of 57.3 ± 12.75 years (range 35–80 years) and a median age 58.5 years. Karnofsky performance status ranged between 70-90%. Forty percent of patients were non smoker. Their main complaint was heamaturea (100%) then triad of heamaturea, burning and necroturea (40%).

As regard disease characteristics, pathological diagnosis of T2a was 7%, T2b was 83% and T3 was 10%. Sixty percent of patients had positive history of Bilharziasis. 63.3% of the included patients were transitional bladder carcinoma, 36.7% were high grade tumor and 33.3% the tumor located at anterior bladder wall.

### Response

#### In group (A)

After phase I of treatment the total response was 73.3% [13 patients (43.3%) had complete response, nine patients (30%) had partial response], seven patients (23.3%) had stationary disease and one patient (3.3%) had disease progression. Patients who had complete and partial response (22 patients) received second phase of treatment and eight patients (26.6%) referred for salvage cystectomy.

After end of treatment, 15 patients (50%) had complete response and we followed them up. Seven patients (23.3%) had partial response, seven patients (23.3%) had stationary disease and one patient (3.3%) had a disease progression. Those patients underwent salvage cystectomy except one patient who refused cystectomy (Table 2).

**Table 2:** Response rate for group A and B after concurrent chemo radiotherapy

	**Complete response**		**Partial response**		**Stationary course**		**Progression**		**Rate of salvage cystectomy**	
	**No.**	**%**	**No.**	**%**	**No.**	**%**	**No.**	**%**	**No.**	**%**
**Group A**										
Phase 1	13	43.3%	9	30%	7	23.3%	1	3.3%	8	26.6%
Phase 2	15	50%	7	23.3%	7	23.3%	1	3.3%	14	46.6%
**Group B**										
Phase 1	27	90%	0	0%	3	10%	0	0%	0	0%
Phase 2	27	90%	0	0%	3	10%	0	0%	1	3.33%

The completeness of transurethral resection, and early tumor stage revealed a significant impact on tumor response. (P- value: 0.012 and 0.021 respectively).

#### In group (B)

After conservative treatment, 27 out of 30 patients were disease free at the time of the cystoscopic reevaluation 4–6 weeks after the end of treatment. This was confirmed by the biopsies taken from the site of the primary tumor. Three patients had residual disease. And they were referred for salvage cystectomy (Table 2).

### Radio-chemotherapy related toxicity

No life threatening acute toxicity was noticed in group (A) due to combined radio-chemotherapy. Grade (G) I anemia occurred in 3 patients (10%), GII occurred in 5 patients (16.6%). G I Leucopenia occurred in 6 patients (20%), GII occurred in 2 patients (6.6%), and grade III leucopenia occurred in one patient (3.3%). GCSF support was not required and no treatment interruption was done. Grade I Thrombocytopenia occurred in one patient (3.3%) and GII occurred in one patient (3.3%). Grade I&II gastrointestinal toxicities were noticed in some patients received radio-chemotherapy. No GIII, or IV gastro intestinal toxicity was detected.

Grade III bladder toxicity was noticed in three patients, and the rest of patients had GI, II bladder toxicity. Those patients were managed by symptomatic treatment. 10 patients experienced increase creatinine level (8 patients were GI, and 2 patients were GII). Those patients renal function improved with I.V fluid and increase oral fluid intake. Seven patients had grade I radiation dermatitis (Table 3 and Table 4).

**Table 3:** Acute toxicities after concurrent chemoradiotherapy for bladder preservation

Acute toxicity*	**I**		**II**		**III**	
	**Group A**	**Group B**	**Group A**	**Group B**	**Group A**	**Group B**
• Anemia	10%	26.7%	16.6%	13.2%	0%	3.3%
• leucopenia	20%	9.9%	6.6%	3.3%	3.3%	3.3%
• Thrombocytopenia	3.3%	16.5%	3.3%	6.6%	0%	0%
• Diarrhea	40%	40%	6.6%	23.3%	0%	3.3%
• Vomiting	43.3%	46.7%	13.3%	46.7%	0%	6.6%
• Proctitis	60%	23.3%	10%	6.6%	0%	0%
• Dysuria	33.3%	36.7%	60%	53.3%	6.6%	6.6%
• Frequency/Urgency	12%	43.3%	20%	36.7%	3.3%	16.5%
• Increase creatinine level	26.6%	6.6%	6.6%	3.3%	0%	3.3%
• Increase bilirubin level	0%	0%	0%	0%	0%	0%
• Increase liver enzymes	0%	13.2%	0%	0%	0%	0%
• Radiation dermatitis	23.3%	76.7%	0%	0%	0%	0%

*(NCI toxicity grading).

**Table 4:** Late toxicities after concurrent chemoradiotherapy for bladder preservation

Late toxicity*	**I**		**II**		**III**		**IV**	
	**Group A**	**Group B**	**Group A**	**Group B**	**Group A**	**Group B**	**Group A**	**Group B**
• Dysuria	13.3%	23.3%	33.3%	6.6%	0%	3.3%	0%	0%
• Frequency	53.3%	23.3%	13.3%	13.2%	0%	6.6%	0%	0%
• Proctitis	16.6%	6.6%	6.6%	0%	0%	0%	0%	0%
• Increase creatinine level	6.6%	3.3%	0%	3.3%	0%	0%	3.3%	0%

*(NCI toxicity grading).

For group (B), the treatment was well tolerated, although anemia grade 3 was observed in one patient and grade 3 frequency occurred in 5 (16.5%) patients, dysurea was reported in 2 (6.6%) patients and bladder capacity was reduced to less than 250 cc in 3 patients (Table 3 and Table 4).

### Recurrence free survival and overall survival

#### For group (A)

The follow up period ranged from 12 to 30 months with median follow up 17 months, after end of treatment 15 patients were free of disease after bladder preservation, and 15 patients did not achieve CR, out of them,14 patients underwent salvage cystectomy, and one patient refused cystectomy.

During the period of follow up, out of the group of preserved bladder, four patients showed disease recurrence. Out of the 14 patients who underwent salvage cystectomy two patients developed disease recurrence. The actuarial disease free survival curve for both groups shows that, the disease free survival at the end of follow up (30 months) was 70% for the preserved bladder group and 75% for the salvage cystectomy group (P- 0.74).

**Figure 1:**
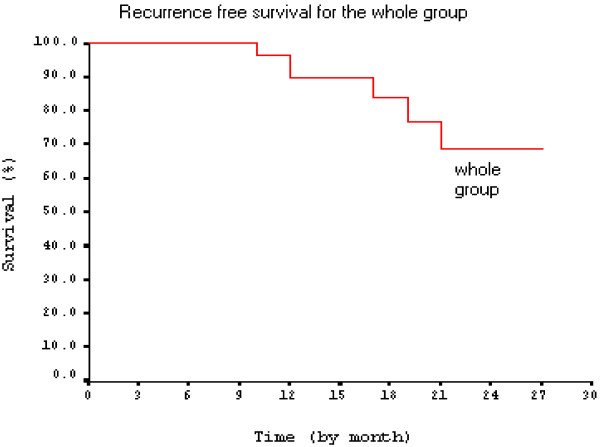
Recurrence free survival for group A.

From the actuarial recurrence free survival curve for the whole group of patients (Figure 1), we noticed that the recurrence free survival at the median follow up (17 months) was 84% and at the end of follow up (30 months) was 70%.

**Figure 2:**
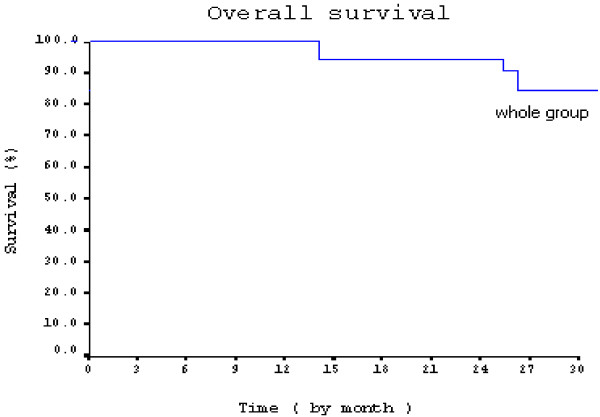
Overall survival curve for group A.

The overall survival for group A at the median follow up 17 months was 95%. Overall survival for the preserved bladder patients at the end of follow up (30 months) was 92% and for the salvage cystectomy patients was 84%. (Figure 2).

#### Group (B)

After conservative treatment 27 out of 30 patients were disease free at the time of the cystoscopic reevaluation 4–6 weeks after the end of treatment which was confirmed by biopsies taken from the primary tumor site. Two patients has residual disease and one patient died after end of treatment due to other cause rather than disease progression.

**Figure 3:**
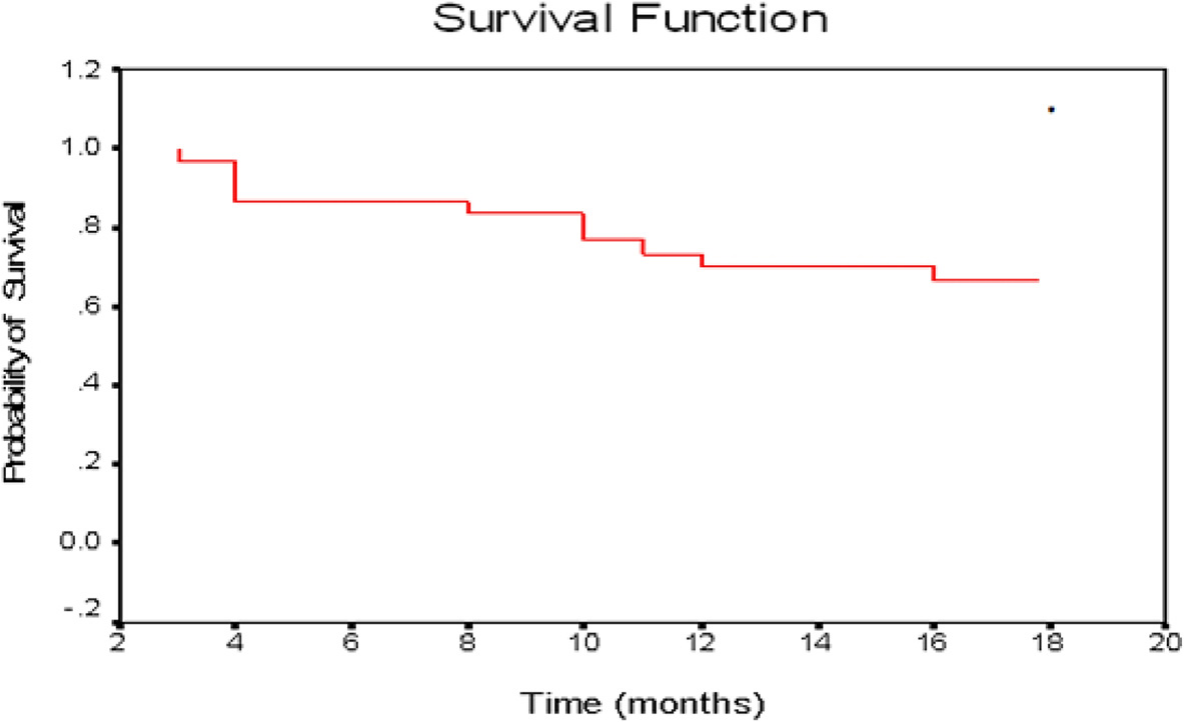
Recurrence free survival for group B.

**Figure 4:**
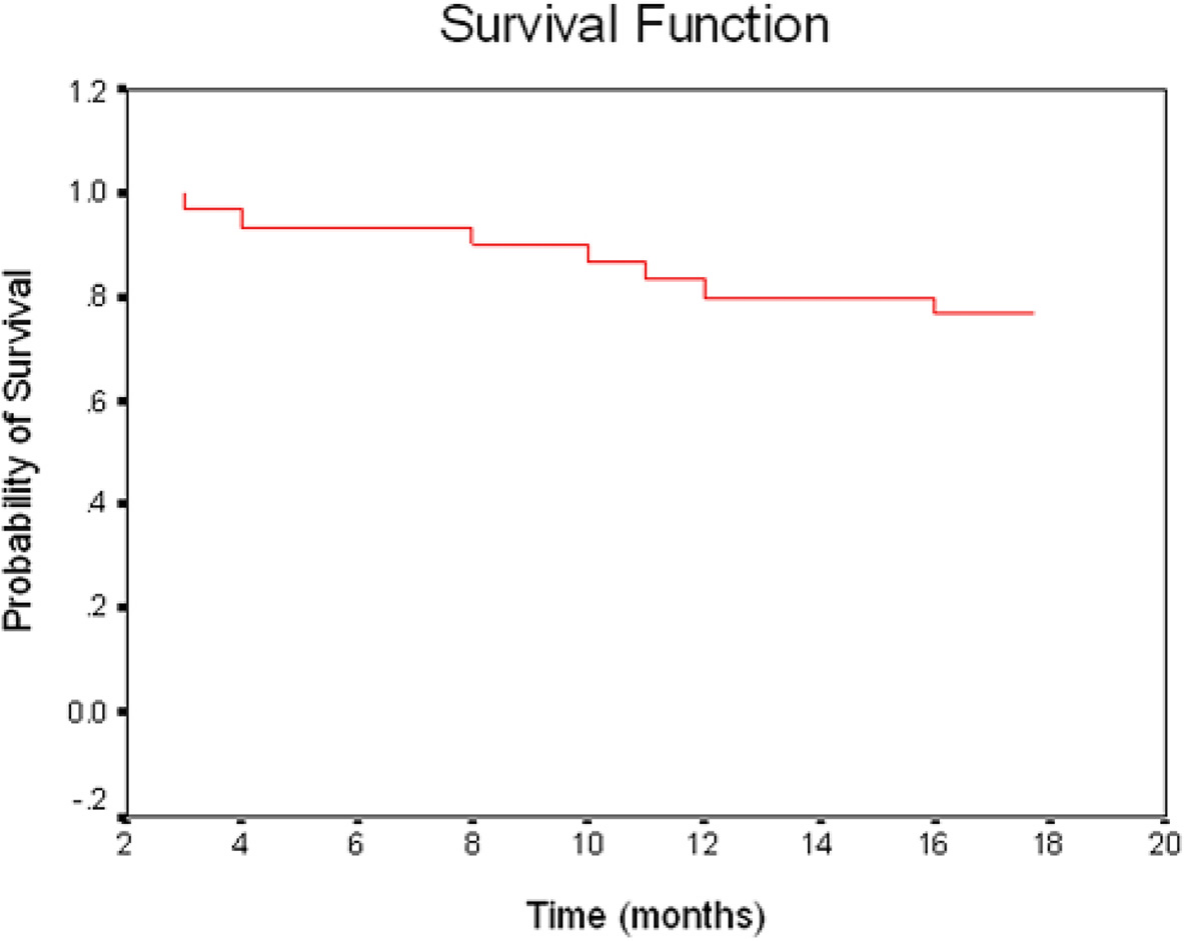
Overall survival curve for group B.

Out of 27 patients, 6 developed locally infiltrating bladder recurrence, and one patient developed distant metastases (to the lung) during 18 months follow up. Salvage cystectomy was done for 3 patients and 3 patients refused cystectomy. Disease free survival (DFS) was 66.67% with mean 14.7 and median 18 months. Overall survival (OAS) was 76.67% with mean 15.93 and median 18 months (Figure 3 and Figure 4).

Three significant prognostic factors were detected for overall survival (OS), performance status with P. value= 0.032, tumor size with P. value=0.000 and residual tumor with P. value=0.000.

Two significant prognostic factors were detected for disease free survival (DFS), tumor size with P. value=0.017 and residual tumor with P. value=0.033.

## Discussion

In group (A) study the age of patients ranged from 35-72 ys with Median 56 years, 24 patients were male (80%) and 6 patients were female (20%). Performance status was represented as following, 23 patients (76.6%) were scale (1) and seven patients (23.3%) were scale (2).

In group (B) the mean age was 57.30 years, median 58.5 and range (35 to 80) years with peak incidence in 7th decade (9 cases) then in 6th decade 7 cases (23.33%) and that was comparable with series of Zaghloul et al., 2002 in which the mean age was (56.24) and the peak of incidence was in 6th and 7th decades [4]. It was also comparable with other western studies, such as Shipley et al study, in which the incidence increases with age and peaks in the sixth and seventh decades of life [5].

Comparison between patient’s criteria of our studies and other series is presented in Table 5.

**Table 5:** Comparison between patients’ number, clinical stages, grades, and tumor type, in our studies and other series

	**Arias, et al., 2000 **[6]	**Caffo et al., 2003 **[8]	**Caffo et al., 2003 **[8]	**Sakr et al., 2003 **[9]	**Soliman et al., 2004 **[10]	**Group A**	**Group B**
Patients number	50	415	16	30	25	30	30
Tumor type	TCC	TCC	TCC	Mixed (TCC & SCC)	TCC	TCC	Mixed
T1	_____	21%	_____	_____	______	_____	______
T2	68%	51%	88.9%	53.4%	48%	36.6%	90%
T3	32%	7%	_____	46.6%	52%	59.9%	10%
T4a	_____	____	11.1%	______	_______	3.3%	_______
GI	10%	______	unknown	______	_______	_____	36.7%
GII	40%	47.5%	unknown	76.6%	60%	60%	33.3%
GIII	50%	52.5%	unknown	23.4%	40%	40%	30%

In group (B), 90% of patients were disease free at the time of cystoscopic reevaluation and this is in agreement with the results of Danesi, et al. 2004 in which complete response was found in 90.3% of included patients. They treated their patients by transurethral resection, protracted intravenous infusion chemotherapy (cisplatin and 5 F.U.), and hyper fractionated radiotherapy [11]. The response in this study is also comparable to that reported by Caffo et al., 2003 [8] who reported a response rate of 100% and they treated their patients in phase I study of gemcitabine and radiotherapy plus cisplatin after transurethral resection as conservative treatment for infiltrating bladder Cancer.

On the other hand response was higher than reported by group (A), in which they used (5-FU & cisplatin), concomitant with twice daily fractionated radiotherapy (accelerated hyperfractionation), the total responders were 73.3% of patients (complete response was in 43.3% and partial response was in 30%).

Rodel et al., 2002, used concurrent radiotherapy and 5-FU & cisplatin. The complete response rate was 72% and Soliman et al., 2004 [10], reported total responders 76% (complete response was in 60% and partial response was in 16%) and he also used concurrent radiotherapy and 5-FU & cisplatin, and lastly group A results was higher than results achieved by Sakr 2003, who reported 37% complete response rate, but he used sequential chemotherapy (3 cycles Gemcitabine and Cisplatin) and radiotherapy [9].

Of concern is that within 18 months of follow up in group (B), 7 of 27 complete responding patients had local recurrence, in addition to 3 patients who did not achieve complete response, they constitute 33.3% of all included patients. This is better than that reported by Danesi D.T., et al., 2004 [11] where local recurrence was 53.5%. The difference may be due to higher number of patients included in their study (72 patients) and longer follow up period (30 months).

Results reported by group (A), showed overall survival 95% for preservation group, and 92%, for primary cystectomy group at median follow up 17 months, and at the end of 30 months follow up, OS were 84%, and 78% respectively. Recurrence free survival for the whole group of patients was 70%. Higher results was reported by Rodel, et al., 2002, who reported 80% disease free survival at 18 months follow up, and also by Soliman et al. 2004, who reported 88.5% disease free survival at the same follow up period.

The overall Survival in group (B) was 76.7% at 18 months, this was different from that reported by Kaufman et al., 2000 [12]. Who reported an actuarial overall survival at three years 83%, the difference may be due to the use of induction treatment by chemoradiation, and patients with complete response received consolidation therapy with the same drugs and combined with twice daily radiation therapy for a total of 20 Gy. Also it was lower than what was reported by group (A), as the overall survival for preservation group and primary cystectomy group at median follow up 17 months were 95% and 92%, respectively. At the end of follow up, 30 months, OS was 84%, and 78% respectively. But it was comparable with results reported by Rodel, et al., 2002, who reported 74%.overall survival at 18 months.

In group (B) three significant prognostic factors were detected for OS, performance status, tumor size and residual tumor, and two significant prognostic factors were detected for DFS, tumor size and residual tumor. It was reported by Rodel, et al., 2002 that disease stage, grade and the extent of TUR were significant factors affecting the response [7]. And it is similar to that reported by group (A) as the completeness of TUR and early stage of the tumor had the strongest impact on the response. Soliman et al., 2004, reported that ureteric obstructive uropathy and bilharzias to be significant prognostic factors [10].

The treatment was well tolerated, although anemia Grade 3 was observed in one patient and Grade 3 cystitis occurred in 5 patients and bladder capacity was reduced to less than 250 c.c. in 3 patients and that was similar with that reported by Caffoo et al., 2003 as grade 3 hematological toxicity was documented in l patient. Also it was comparable to group (A) in which grade 3 leucopenia was found in one patient, 2 patients (6.6%) had GIII dysuria, and one patient (3.3%) had GIII frequency. More hematological toxicity reported by Kaufman, et al., 2000, who reported 21% of patients developed GIII- IV hematological toxicity [12], and Rodel, et al., 2002, as 23% of cases developed GIII leucopenia, and 4% had GIII anemia [7]. Actually, bladder cancer recurrence usually happen early during the follow-up period according to other trials With a median follow-up of 38 months in Slaton et al. study, 25% of patients experienced recurrences, with a median time to recurrence of 12 months. The 4 most common sites of recurrence (in decreasing order of incidence) were the lung, pelvis, bone, and liver. All recurrences in patients with pT2 or pT3 disease occurred within 24 months [13]. A concern to bladder preservation protocols is the possibility of local recurrence of the same disease or new disease. In a series from MGH, 26% of patients experienced a local recurrence at a median of 2.1 years after definitive treatment [14]. The median follow up in our study was nearly equal to previous studies done at the same geographic area, with nearly the same tumor biology, as the median follow up was 2 years in Abbas, etal, study [15]. Even we closed our studies; our patients still under regular follow up by our institutions of protocol.

### Limitation of the study

The number of included patient was relatively small, because most of the patients present when bladder preservation is not possible at their stage of presentation, in addition to lack of referral of early cases from community urologist for bladder preservation. Even our patients had a better quality of life compared to our previous experience with those treated without bladder preservation protocol; we did not add this as a solid result, because our study was not double arm study.

## Conclusion

From our study we conclude that bladder preservation is a valid option for bladder cancer treatment. And it should be of higher priority especially in areas where bladder cancer is more common.

Further trials are needed to evaluate different strategies for combined modality therapy. The next generation of studies should incorporate prognostic and predictive biomarkers in bladder Cancer. It is hoped that biomarkers, as well as predicting survival will also have value in predicting those who will respond to chemoradiation therapy and keep their bladder. This would allow selection of patient for bladder preservation at the time of diagnosis.

Adjuvant chemotherapy and hyperfractionation must be evaluated in further trials in attempt to improve disease free survival and overall survival.

Lastly, phase trials III must be done to compare between bladder preservation protocols and total cystectomy for better survival for bladder cancer patients with better improvement in patient quality of life.

## Competing interests

The authors declare that they have no competing interests.

## Authors’ contributions

The first and third authors followed the patients and collected the data of study (B). The second and fourth authors followed the patients and collected the date of study (A). All authors contributed equally in writing the paper, and preparing tables and diagrams. The third author carried the responsibility of paper submission. All authors read and approved the final manuscript.
